# Cause-specific mortality after endovascular treatment for anterior vs. posterior circulation large vessel occlusion: a multicenter cohort study

**DOI:** 10.3389/fneur.2026.1948536

**Published:** 2026-10-26

**Authors:** Miao Li, Yue Wan, Yi Mo, Jingwei Li, Xiaoli Zhang, Xiongjun He, Hanlin Chen, Mingxuan Huang, Jinghan Guo, Feng Gao, Dapeng Mo, Ning Ma, Zhongrong Miao, Anxin Wang, Yiming Deng

**Affiliations:** 1 Interventional Neuroradiology, Department of Neurology, Beijing Tiantan Hospital, Capital Medical University, Beijing, China; 2 China National Clinical Research Center for Neurological Diseases, Beijing, China; 3 Department of Neurology, The Third People’s Hospital of Hubei Province, Wuhan, China; 4 Department of Neurosurgery, The First People’s Hospital of Changzhou, Changzhou, China; 5 Department of Neurology of Suzhou Hospital, Affiliated Hospital of Medical School, Nanjing University, Suzhou, China; 6 Department of Epidemiology, Beijing Neurosurgical Institute, Beijing Tiantan Hospital, Capital Medical University, Beijing, China; 7 Department of Neurology, Shenzhen Hospital, Southern Medical University, Guangzhou, China

**Keywords:** acute ischemic stroke, cause-specific mortality, competing risks, endovascular treatment, large vessel occlusion

## Abstract

**Background:**

Acute ischemic stroke due to large vessel occlusion (AIS-LVO) carries substantial mortality despite endovascular treatment (EVT). Whether causes of death differ between anterior (AVO) and posterior (PVO) circulation occlusions is poorly characterized. We compared cause-specific 90-day mortality between patients with AVO and PVO treated with EVT.

**Methods:**

In this multicenter retrospective cohort, consecutive patients with AIS-LVO who underwent EVT at 111 hospitals in China (ANGEL-ACT registry [Endovascular Treatment Key Technique and Emergency Workflow Improvement of acute ischemic stroke]) were included, and adjudicated causes of death were grouped into prespecified categories. Cause-specific mortality was assessed in the complete analytic cohort using the Fine-Gray subdistribution hazard model, with survivors censored at 90 days and deaths from other causes treated as competing events. Cumulative incidence functions were estimated with the Aalen-Johansen estimator.

**Results:**

Among 1,671 eligible patients (1,300 AVO, 371 PVO), 228 deaths (13.6%) occurred within 90 days. All-cause mortality was higher in PVO than AVO (19.4% vs. 12.0%). Cerebral edema-related death was significantly more frequent in PVO than AVO (90-day cumulative incidence 9.16% vs. 4.15%; adjusted subdistribution hazard ratio [sHR] 0.47, 95% CI 0.30–0.73, *p* = 0.001; Gray’s test *p* < 0.001). Fatal hemorrhagic transformation was numerically more frequent in AVO but did not reach statistical significance in the complete cohort (3.77% vs. 1.89%; adjusted sHR 1.76, 95% CI 0.78–3.98, *p* = 0.17; Gray’s test *p* = 0.076), consistent across all sensitivity analyses. Pneumonia-related (adjusted sHR 0.34, 95% CI 0.17–0.68, *p* = 0.003) and recurrent stroke-related (adjusted sHR 0.29, 95% CI 0.10–0.86, *p* = 0.026) deaths were also more frequent in PVO; the pneumonia association remained significant after Bonferroni correction, whereas the recurrent stroke association did not.

**Conclusion:**

Fatal complications after EVT differ by occlusion territory. Posterior circulation occlusion is independently associated with a higher risk of death from cerebral edema, pneumonia, and recurrent stroke, although the recurrent stroke association did not survive Bonferroni correction. Hemorrhagic transformation accounted for a larger share of anterior circulation deaths, but this did not correspond to a statistically significant increase in risk in the complete cohort.

## Introduction

Acute ischemic stroke (AIS) secondary to large vessel occlusion (LVO) is associated with persistently high mortality and severe long-term disability, even among patients who receive timely reperfusion therapy (1, 2). Endovascular treatment (EVT) has been established as the standard of care for patients with AIS-LVO presenting within the eligible therapeutic window, as it significantly improves functional outcomes compared with best medical management alone (3–5). Despite these advances, 90-day mortality rates remain substantial, ranging from 15 to 30% in both real-world registries and randomized controlled trials (6, 7). Elucidating the specific causes of death in this patient population is therefore critical to identifying high-risk individuals and developing targeted interventions to improve survival.

Anterior circulation LVO (AVO) and posterior circulation LVO (PVO) differ fundamentally in the vascular territories affected, collateral circulation, clinical presentation, and prognosis (8, 9). Previous studies have shown that patients with PVO face a higher risk of adverse outcomes, attributed to delayed diagnosis, greater baseline stroke severity, and the vulnerability of the brainstem to ischemic injury (10, 11). In contrast, AVO is more strongly associated with cardioembolic stroke etiology and with an elevated risk of hemorrhagic transformation after reperfusion (12). Despite these well-documented differences, few studies have systematically compared the specific causes of death between patients with AVO and PVO treated with EVT. Most mortality analyses from major EVT trials have reported overall mortality rather than cause-specific outcomes (13, 14), so whether the distribution of fatal complications varies by occlusion location remains unclear.

Accordingly, this study aimed to characterize 90-day cause-specific mortality in patients with AIS-LVO treated with EVT, stratified by anterior versus posterior circulation occlusion site, using a competing-risk framework applied to the complete cohort. Using data from the nationwide ANGEL-ACT registry, we further sought to identify independent associations between occlusion location and cause-specific mortality after adjustment for predefined potential confounders.

## Methods

### Study population

This study is a retrospective analysis of data from the ANGEL-ACT registry (Endovascular Treatment Key Technique and Emergency Workflow Improvement of acute ischemic stroke), a prospective, multicenter, nationwide clinical registry conducted at 111 hospitals in China between November 2017 and March 2019. The registry consecutively enrolled adult patients with AIS due to LVO who underwent EVT, and collected baseline clinical characteristics, procedural parameters, and longitudinal clinical outcomes. The registry’s full inclusion and exclusion criteria have been described previously (15).

Of the 1793 patients enrolled in the registry, inclusion criteria for the present analysis were: (1) age ≥18 years; (2) LVO confirmed by imaging; and (3) treatment with any EVT modality, including intra-arterial thrombolysis, mechanical thrombectomy, angioplasty, and permanent stent placement. Exclusion criteria were: (1) missing 90-day follow-up data (*n* = 77); (2) missing data on the confirmed cause of death (*n* = 45). In total, 1,671 patients were eligible for this analysis (Figure 1).

**Figure 1:**
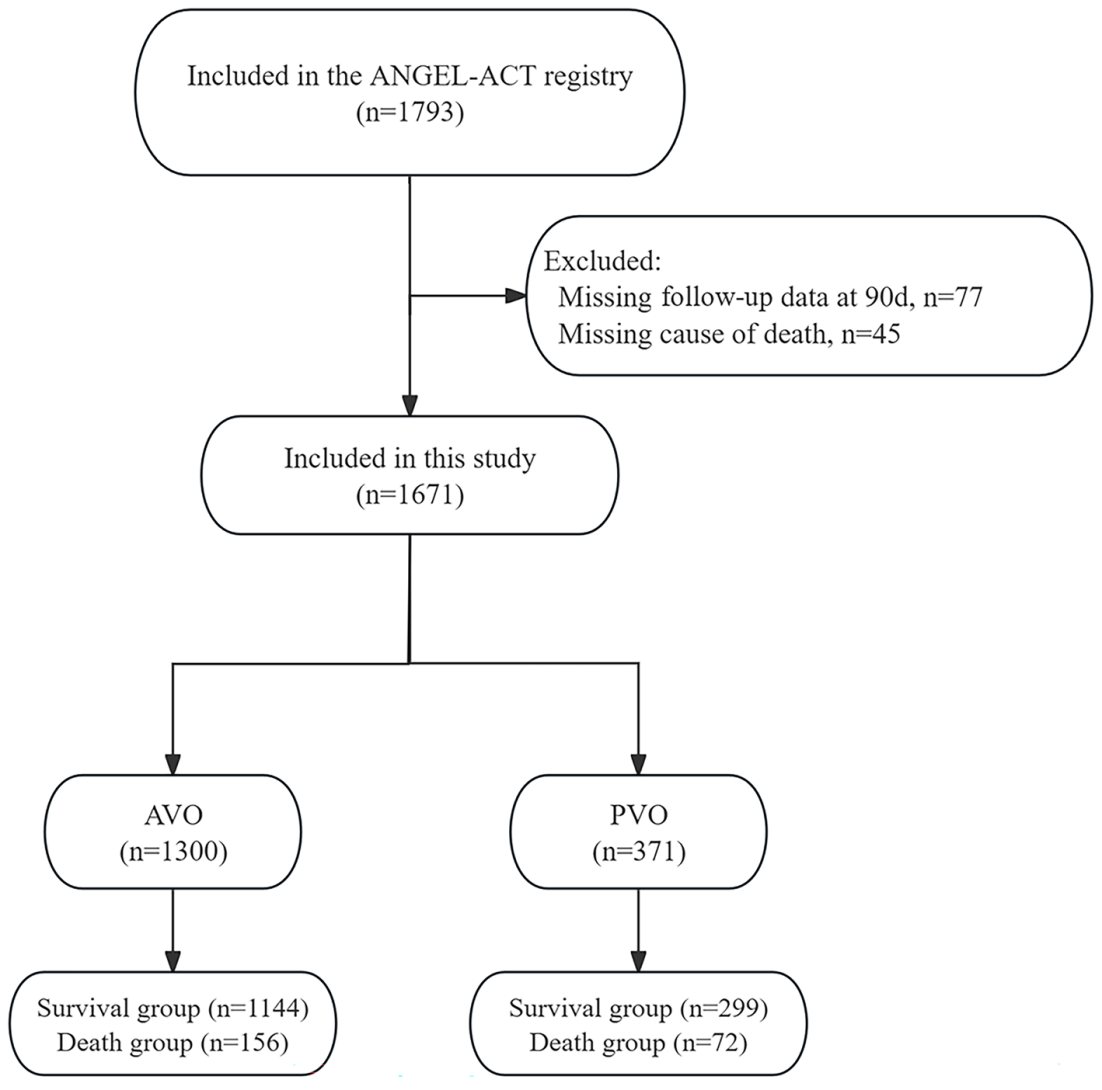
Study flow diagram. Of 1793 patients enrolled in the ANGEL-ACT registry, 122 were excluded: 77 with missing 90-day follow-up and 45 with an unconfirmed cause of death. All 45 patients excluded for an unconfirmed cause of death had died; 37 of these had anterior circulation occlusion and 8 posterior circulation occlusion. The remaining 1,671 patients comprised 1,300 with anterior circulation occlusion (AVO) and 371 with posterior circulation occlusion (PVO). Ninety-day survival and death counts are shown for each group.

All 45 patients excluded for an unconfirmed cause of death had in fact died; their deaths were documented in the registry’s fatality log, but the adjudication committee was unable to assign a cause. The registry recorded 273 deaths among the 1793 enrolled patients: 228 with an adjudicated cause and 45 without. Occlusion territory was recorded for the 45 patients: 37 (82.2%, 95% CI 71.1–93.4) had anterior circulation occlusion and 8 (17.8%) posterior circulation occlusion. Cause-specific analyses are therefore restricted to the 1,671 patients with an adjudicated cause, and the full exclusion flow is reported in Figure 1. All-cause mortality is reported both for the 1,671-patient analytic cohort and, as a sensitivity analysis, for the 1716 patients who survived to 90 days or had a documented death (that is, before exclusion of the 45 deaths of undetermined cause).

### Definition of circulation territory and occlusion site

Occlusion location was recorded at the vessel level and mapped to circulation territory as follows. Anterior circulation occlusion comprised occlusion of the intracranial internal carotid artery (ICA), the first segment of the middle cerebral artery (M1), or the second segment of the middle cerebral artery (M2). Posterior circulation occlusion comprised vertebrobasilar occlusion (V-BA), defined as occlusion of the basilar artery or a dominant vertebral artery. Posterior cerebral artery occlusion was not included in the PVO definition in this analysis.

### Data collection

Standardized data collection captured predefined elements: baseline clinical characteristics (age, sex, medical history, baseline National Institutes of Health Stroke Scale [NIHSS] score, and stroke subtype by TOAST criteria), periprocedural and procedural parameters, periprocedural complications, and 90-day mortality with cause of death.

For all included patients, the cause and timing of death were verified by one of two standardized approaches: review of serious adverse event (SAE) forms by the registry’s dedicated SAE Committee for cases with documented fatal SAEs, or independent review of hospital discharge summaries for cases without a recorded fatal SAE. Discharge summaries were evaluated independently by two trained investigators, and discrepancies were resolved by consensus. Mortality events were further categorized as in-hospital or post-discharge. For cases with incomplete data, supplementary clinical information was obtained by contacting the treating physician responsible for the patient’s care at the time of death, including general practitioners or nursing home clinicians for patients who died at home or in long-term care facilities. All cause-of-death adjudications were performed by two independent neurologists blinded to occlusion territory, with disagreements resolved by a third senior adjudicator.

### Outcome definitions

The primary outcome was the between-group difference in 90-day cause-specific mortality between the AVO and PVO groups, assessed in the complete analytic cohort. The secondary outcome was the between-group difference in the subdistribution hazard of each individual cause of death. Drawing on prior literature on causes of death after endovascular treatment (16–19) and on the available clinical, imaging, and laboratory data, causes of death were classified into the following prespecified categories:

1. Cerebral edema: Adjudicated when neurological deterioration occurred in the setting of clinical and radiographic findings of space-occupying cerebral edema that was determined to be the direct cause of death.
2. Hemorrhagic transformation: Defined as imaging findings consistent with Heidelberg Bleeding Classification classes 1–3 accompanied by neurological deterioration (an increase in NIHSS score of ≥4 points), with the complication adjudicated as the direct cause of death by the clinical events committee or treating physician.
3. Pneumonia: Diagnosed when infection-related systemic signs, respiratory symptoms, or compatible chest imaging findings were present and pneumonia was confirmed as the direct cause of death by the adverse event committee or treating physician.
4. Withdrawal of care due to poor neurological prognosis: Applied when life-sustaining interventions were withheld or discontinued because of a lack of improvement in the index stroke, in the absence of other identifiable causes of death.
5. Recurrent stroke: Defined as a new ischemic stroke (in the same or another vascular territory) that resulted in neurological deterioration and subsequent withdrawal of life-sustaining treatment, with the event adjudicated as the primary cause of death.
6. Atrial fibrillation-related death: Adjudicated when death occurred in the setting of a documented arrhythmia-related hemodynamic or embolic event, specifically fatal arrhythmia (sustained ventricular tachyarrhythmia or asystole) or systemic embolization attributable to atrial fibrillation, after other causes had been excluded. A history of atrial fibrillation alone, without a documented acute arrhythmic or embolic terminal event, was not sufficient for this category.
7. Neurogenic causes: A composite category comprising cerebral edema, hemorrhagic transformation, recurrent stroke, and withdrawal of care due to poor neurological prognosis.
8. Cardiogenic causes: A composite category comprising death related to atrial fibrillation, heart failure, acute pulmonary embolism, and acute myocardial infarction.
9. Other causes: A composite category comprising death due to pneumonia, other infectious diseases, gastrointestinal hemorrhage, and all other non-neurogenic, non-cardiogenic etiologies.

Adjudication hierarchy for fatal hemorrhagic transformation versus fatal cerebral edema. The Heidelberg Bleeding Classification provides an anatomical description of post-reperfusion hemorrhage; it does not establish that the hemorrhage, rather than the underlying infarction and its associated edema, caused death. We therefore applied a prespecified adjudication hierarchy. For each death in which both hemorrhage and space-occupying edema were present, the adjudicating neurologists reviewed serial imaging and assigned the direct cause of death in the following order of precedence: (1) hemorrhage with documented expansion on serial imaging; (2) hemorrhage with hematoma-related mass effect, midline shift, or ventricular compression attributable to the hematoma rather than to the infarct; (3) in the absence of (1) or (2), infarct-related swelling with ventricular compression, hydrocephalus, or herniation attributable to the infarct and its edema. Hemorrhages of Heidelberg classes 1a, 1b, and 1c, which have absent or limited hemorrhagic mass effect and frequently coexist with extensive infarct-related edema, were classified as the direct cause of death only when criteria (1) or (2) were met. Cases in which serial imaging and the clinical course could not separate the two mechanisms were adjudicated as mixed or indeterminate and were assigned to neither the hemorrhagic transformation nor the cerebral edema category. An increase in NIHSS of ≥4 points established clinical deterioration but was not itself treated as evidence of mechanism.

### Statistical analysis

This retrospective observational analysis included 1,671 patients with AIS-LVO treated with EVT. Continuous variables are presented as median with interquartile range (IQR) and categorical variables as counts with percentages. Baseline and procedural characteristics were compared between groups using the Mann–Whitney *U* test for continuous variables and the Pearson χ^2^ test or Fisher’s exact test for categorical variables, as appropriate.

To assess the association between occlusion territory (AVO vs. PVO, with PVO as the reference) and each cause-specific death, we applied the Fine-Gray subdistribution hazard model to the complete analytic cohort of 1,671 patients. Survivors were censored at 90 days, and deaths from causes other than the cause of interest were treated as competing events. Results are expressed as subdistribution hazard ratios (sHRs) with 95% confidence intervals (CIs). Cumulative incidence functions (CIFs) for each cause-specific death were estimated with the Aalen-Johansen estimator, again treating deaths from other causes as competing events, and differences in CIFs between groups were compared with Gray’s test. Ninety-day cumulative incidences are reported as percentages with 95% CIs.

Multivariable analysis was adjusted for the following prespecified potential confounders: age, sex, systolic blood pressure, type of anesthesia, history of atrial fibrillation, successful recanalization (modified Thrombolysis in Cerebral Infarction grade 2b–3), and smoking status. Covariates were selected *a priori* on the basis of clinical relevance and baseline imbalance between groups. Because stroke mechanism and baseline stroke severity have been identified as potential confounders in prior literature, a second, extended model further adjusted for TOAST classification, baseline NIHSS score, onset-to-admission time, door-to-puncture time, puncture-to-recanalization time, and pretreatment intravenous thrombolysis. This extended model is reported as a sensitivity analysis, because baseline NIHSS and TOAST classification lie on the causal pathway between occlusion territory and fatal complications; adjusting for them may therefore attenuate a genuine territory effect.

Four sensitivity analyses were performed. Model 1 was adjusted for age and sex only. Model 2 was restricted to patients without a history of atrial fibrillation. Model 3 was restricted to patients who achieved successful recanalization. Model 4 excluded deaths attributed to withdrawal of care due to poor neurological prognosis, because decisions to withdraw life-sustaining treatment vary across centers and may mask the underlying neurological injury. To account for multiple testing, Bonferroni correction and Benjamini-Hochberg false discovery rate adjustment were both applied to the primary outcomes, and the adjusted *p* values (Bonferroni_P and BH_P) are reported accordingly. A two-sided *p* value <0.05 was considered statistically significant. Individual causes with fewer than ten events were not modeled separately and were reported only within their composite category, because the Fine-Gray model produces unstable or non-estimable coefficients at these event counts; gastrointestinal hemorrhage, for which no events occurred in the posterior circulation group, produced complete separation. All analyses were conducted in R (version 4.5.2, R Foundation for Statistical Computing, Vienna, Austria) with the cmprsk and survival packages.

## Results

### Patient characteristics

Of the 1793 patients enrolled in the registry, 122 were excluded (77 with missing 90-day follow-up and 45 with an unconfirmed cause of death), leaving 1,671 patients eligible for analysis (Figure 1). Baseline characteristics are compared in Table 1. Patients with PVO were younger (median 64.0 vs. 66.0 years, *p* = 0.013) and more frequently male (77.1% vs. 63.2%, *p* < 0.001). Compared with the AVO group, the PVO group had a higher prevalence of hypertension and dyslipidemia but a substantially lower prevalence of atrial fibrillation (14.3% vs. 35.2%, *p* < 0.001). Stroke severity on admission was greater in PVO (median NIHSS score 20.0 vs. 15.0, *p* < 0.001), and general anesthesia was used more often (55.8% vs. 35.4%, *p* < 0.001). Large artery atherosclerosis was the most common etiology in both groups, but was more frequent in PVO (65.5% vs. 47.2%), whereas cardioembolism was more frequent in AVO (37.3% vs. 18.3%). Vessel-level occlusion site was recorded for 1,632 patients. Among patients with AVO, occlusion was located at the ICA in 415 (31.9%), M1 in 722 (55.5%), and M2 in 149 (11.5%). Among patients with PVO, occlusion was vertebrobasilar in 346 (93.3% of the PVO group); the remaining 25 PVO patients had no vessel-level site recorded (Table 1).

**Table 1:** Baseline characteristics of patients with AVO and PVO.

Characteristic	AVO (*n* = 1,300)	PVO (*n* = 371)	*p*-value
Age, y; median (IQR)	66.0 (56.0–73.0)	64.0 (55.0–71.0)	0.0134
Male sex, *n* (%)	822 (63.2)	286 (77.1)	<0.0001
History of hypertension, *n* (%)	708 (54.5)	255 (68.7)	<0.0001
History of diabetes mellitus, *n* (%)	227 (17.5)	79 (21.3)	0.108
History of dyslipidemia, *n* (%)	103 (7.9)	49 (13.2)	0.0025
History of atrial fibrillation, *n* (%)	457 (35.2)	53 (14.3)	<0.0001
History of stroke, *n* (%)	246 (18.9)	83 (22.4)	0.1617
History of ICH, *n* (%)	13 (1.0)	5 (1.3)	0.5703
History of CHD, *n* (%)	189 (14.5)	57 (15.4)	0.7545
Prestroke mRS score, *n* (%)^a^			0.4613
0	1,125 (86.5)	320 (86.3)	
1	149 (11.5)	47 (12.7)	
2	25 (1.9)	4 (1.1)	
Cigarette smoking, *n* (%)			0.0028
Never smoker	796 (61.2)	191 (51.5)	
Ex-smoker	88 (6.8)	35 (9.4)	
Current smoker	416 (32.0)	145 (39.1)	
History of drinking, *n* (%)			0.0256
Never drinker	748 (57.5)	182 (49.1)	
Occasional drinker	351 (27.0)	120 (32.3)	
Current drinker	167 (12.8)	54 (14.6)	
Previous drinker	34 (2.6)	15 (4.0)	
Onset-to-admission time, min, median (IQR)^b^	160.0 (70.0–290.0)	125.0 (40.0–256.0)	0.0001
SBP, mmHg; median (IQR)	145.0 (130.0–160.0)	150.0 (137.0–165.0)	<0.0001
NIHSS score, median (IQR)^c^	15.0 (11.2–19.0)	20.0 (11.0–34.0)	<0.0001
ASPECTS, median (IQR)^d^	9.0 (7.0–10.0)	9.0 (7.0–10.0)	0.7821
Stroke subtype by TOAST criteria, *n* (%)			<0.0001
Large artery atherosclerosis	613 (47.2)	243 (65.5)	
Cardioembolism	485 (37.3)	68 (18.3)	
Other or unknown etiology	202 (15.5)	60 (16.2)	
Prior use of antiplatelet agents, *n* (%)	209 (16.1)	75 (20.2)	0.0729
Prior use of anticoagulants, *n* (%)	11 (0.8)	5 (1.3)	0.3704
Intravenous thrombolysis pretreatment, *n* (%)	386 (29.7)	97 (26.1)	0.2061
Types of anesthesia, *n* (%)			<0.0001
Local anesthesia only	609 (46.8)	119 (32.1)	
General anesthesia	460 (35.4)	207 (55.8)	
Local anesthesia plus sedation	231 (17.8)	45 (12.1)	
Tandem stenosis/occlusion, *n* (%)	283 (21.8)	71 (19.1)	0.3067
Thrombectomy pass, median (IQR)	2.0 (1.0–3.0)	1.0 (1.0–2.0)	<0.0001
Intraprocedural administration of heparin, *n* (%)	627 (48.2)	192 (51.8)	0.2552
Intraprocedural administration of Tirofiban, *n* (%)	617 (47.5)	230 (62.0)	<0.0001
DTP, min, median (IQR)^e^	120.0 (80.0–171.8)	132.0 (89.0–195.0)	0.0082
PRT, min, median (IQR)^f^	83.0 (51.0–127.0)	93.0 (59.5–139.5)	0.0112
Successful recanalization, *n* (%)	1,188 (91.4)	330 (88.9)	0.1826
Complete recanalization, *n* (%)	900 (69.2)	222 (59.8)	0.0009
Complications within 2 h, *n* (%)	201 (15.5)	25 (6.7)	<0.0001
Reperfusion injury-induced ICH, *n* (%)	92 (7.1)	7 (1.9)	0.0003
Vascular reocclusion, *n* (%)	70 (5.4)	13 (3.5)	0.1819
Vasospasm, *n* (%)	20 (1.5)	0	0.0119
Vascular dissection, *n* (%)	13 (1.0)	8 (2.2)	0.1076
Vascular perforation-induced hemorrhage, *n* (%)	5 (0.4)	1 (0.3)	1
Vascular perforation-induced SAH, *n* (%)	18 (1.4)	4 (1.1)	0.7996
Any ICH within 24 h, *n* (%)^g^	328 (25.2)	51 (13.7)	<0.0001
Symptomatic ICH within 24 h, *n* (%)^h^	97 (7.5)	11 (3.0)	0.0031
Vessel-level occlusion site, *n* (%)			<0.001
ICA	415 (31.9)	0	
M1	722 (55.5)	0	
M2	149 (11.5)	0	
V-BA	0	346 (93.3)	
Not recorded	14 (1.1)	25 (6.7)	

AVO, anterior circulation large-vessel occlusion; PVO, posterior circulation large-vessel occlusion; IQR, interquartile range; ICH, intracerebral hemorrhage; CHD, coronary heart disease; mRS, modified Rankin Scale; SBP, systolic blood pressure; NIHSS, National Institutes of Health Stroke Scale; ASPECTS, Alberta Stroke Program Early CT Score; TOAST, Trial of Org 10,172 in Acute Stroke Treatment; DTP, door-to-puncture time; PRT, puncture-to-recanalization time; ICA, intracranial internal carotid artery; M1, first segment of the middle cerebral artery; M2, second segment of the middle cerebral artery; V-BA, vertebrobasilar.

Continuous variables are presented as median (IQR) and compared with the Mann–Whitney *U* test; categorical variables are presented as n (%) and compared with the Pearson chi-squared test or Fisher’s exact test as appropriate.

Missing data (*n*): ^a^prestroke mRS score, 1; ^b^onset-to-admission time, 38; ^c^baseline NIHSS score, 8; ^d^ASPECTS, 11; ^e^door-to-puncture time, 132; ^f^puncture-to-recanalization time, 1; ^g^any ICH within 24 h, 1; ^h^symptomatic ICH within 24 h, 60. Vessel-level occlusion site was not recorded in 39 patients (14 AVO, 25 PVO) and is shown as a separate row. Percentages are calculated on patients with available data.

The 122 excluded patients formed two groups: 77 lost to follow-up and 45 whose cause of death could not be confirmed. The two groups were similar in baseline characteristics, including stroke severity (median NIHSS 20.0 in both) and occlusion territory (anterior circulation in 76.6 and 82.2%, respectively; *p* = 0.617), and differed only in the rate of successful recanalization, which was lower among patients lost to follow-up (74.0% vs. 91.1%, *p* = 0.040) (Supplementary Table 1).

### Cause-specific mortality in the complete cohort

Ninety-day cause-specific cumulative incidences and adjusted subdistribution hazard ratios are presented in Table 2. The distribution of causes among the 228 deaths is shown in Figure 2, and the corresponding cumulative incidence curves in Figure 3.

**Table 2:** Primary analysis: Cause-specific 90-day mortality comparing anterior versus posterior circulation occlusion (Fine-Gray model, complete cohort, *N* = 1,671).

Cause of death	AVO (*n* = 1,300) 90-day CIF, % (95% CI)	PVO (*n* = 371) 90-day CIF, % (95% CI)	Gray’s test P	Unadjusted sHR (95% CI)	Adjusted sHR (95% CI)^a^	*p*-value	Bonferroni P	BH P
Cerebral edema	4.15 (3.07–5.24)	9.16 (6.22–12.11)	<0.001	0.44 (0.29–0.68)	0.47 (0.30–0.73)	0.001	0.009	0.004
Hemorrhagic transformation	3.77 (2.73–4.81)	1.89 (0.50–3.27)	0.076	2.02 (0.91–4.45)	1.76 (0.78–3.98)	0.17	1.000	0.267
Pneumonia	1.31 (0.69–1.93)	3.50 (1.63–5.38)	0.005	0.37 (0.18–0.76)	0.34 (0.17–0.68)	0.003	0.030	0.010
Recurrent stroke	0.38 (0.05–0.72)	1.62 (0.33–2.90)	0.010	0.24 (0.07–0.78)	0.29 (0.10–0.86)	0.026	0.286	0.048
Atrial fibrillation	0.69 (0.24–1.14)	0.54 (0.00–1.29)	0.747	1.29 (0.28–5.94)	1.17 (0.28–4.99)	0.83	1.000	0.830
Withdrawal of care	0.38 (0.05–0.72)	1.35 (0.17–2.52)	0.034	0.28 (0.08–0.98)	0.20 (0.06–0.71)	0.013	0.143	0.036
Neurogenic causes^b^	8.69 (7.12–10.27)	14.02 (10.33–17.70)	0.003	0.61 (0.44–0.84)	0.59 (0.42–0.82)	0.002	NA	NA
Cardiogenic causes^c^	1.77 (1.05–2.49)	1.62 (0.32–2.91)	0.844	1.09 (0.45–2.69)	0.78 (0.31–1.93)	0.59	NA	NA
Other causes^d^	1.54 (0.87–2.21)	3.77 (1.83–5.72)	0.007	0.40 (0.20–0.80)	0.34 (0.17–0.67)	0.002	NA	NA

AVO, anterior circulation large-vessel occlusion; PVO, posterior circulation large-vessel occlusion; CIF, cumulative incidence function; sHR, subdistribution hazard ratio; CI, confidence interval; BH, Benjamini-Hochberg.

^a^Adjusted for age, sex, systolic blood pressure, type of anesthesia, history of atrial fibrillation, successful recanalization, and smoking status. The reference group is posterior circulation large-vessel occlusion (PVO); sHRs <1 indicate lower risk in AVO.

Model details: The Fine-Gray subdistribution hazard model was fitted in the complete analytic cohort of 1,671 patients, with survivors censored at 90 days and deaths from other causes treated as competing events. Ninety-day cumulative incidences were estimated with the Aalen-Johansen estimator; Gray’s test was used to compare cumulative incidence functions. Bonferroni P and BH P denote p values corrected for multiple comparisons across the 11 individual causes of death using the Bonferroni and Benjamini-Hochberg methods, respectively. The three composite categories aggregate those same 11 causes and therefore describe the same deaths; admitting them to the correction family would count every death more than once, so they are reported without multiplicity adjustment and are marked NA.

Individual causes with fewer than ten events (heart failure, *n* = 9; acute pulmonary embolism, *n* = 5; myocardial infarction, *n* = 4; other infectious diseases, *n* = 2; gastrointestinal hemorrhage, *n* = 2) were not modeled separately and are reported only within their composite categories, because the Fine-Gray model yields unstable or non-estimable coefficients at these event counts; gastrointestinal hemorrhage, for which no events occurred in the posterior circulation group, produced complete separation.

**Figure 2:**
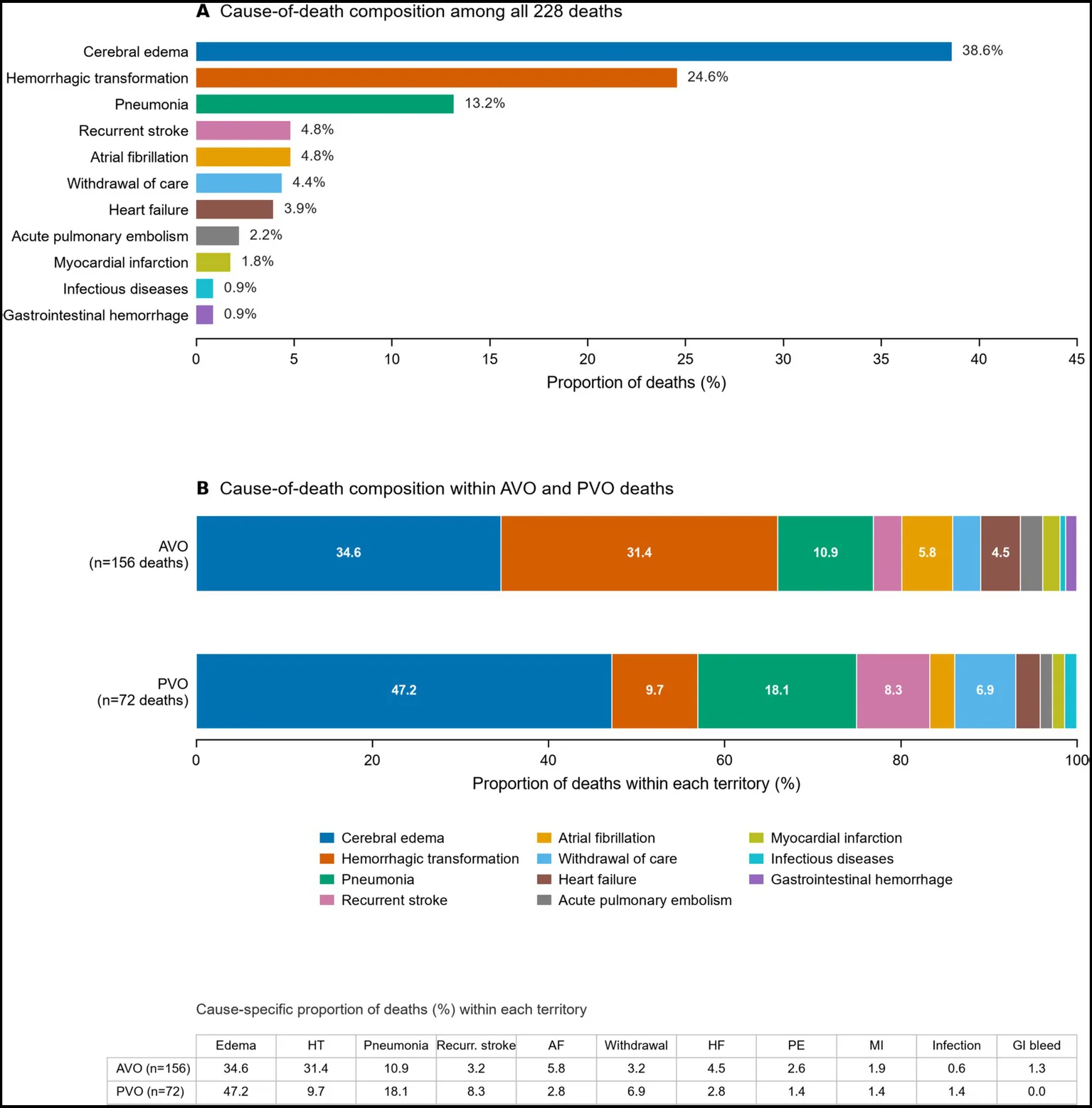
Composition of causes of death among the 228 patients who died within 90 days. **(A)** Distribution of causes of death in the overall deceased population. **(B)** Composition of causes of death within the AVO and PVO groups. Percentages in both panels are calculated among deaths within the relevant group, not among all treated patients; they describe how deaths are distributed across causes and do not estimate the risk of dying from a given cause. Bars are annotated with percentage values for causes accounting for ≥4% of deaths within a group; exact values for all causes are given in the table at the foot of the figure. AVO, anterior circulation large-vessel occlusion; PVO, posterior circulation large-vessel occlusion.

**Figure 3:**
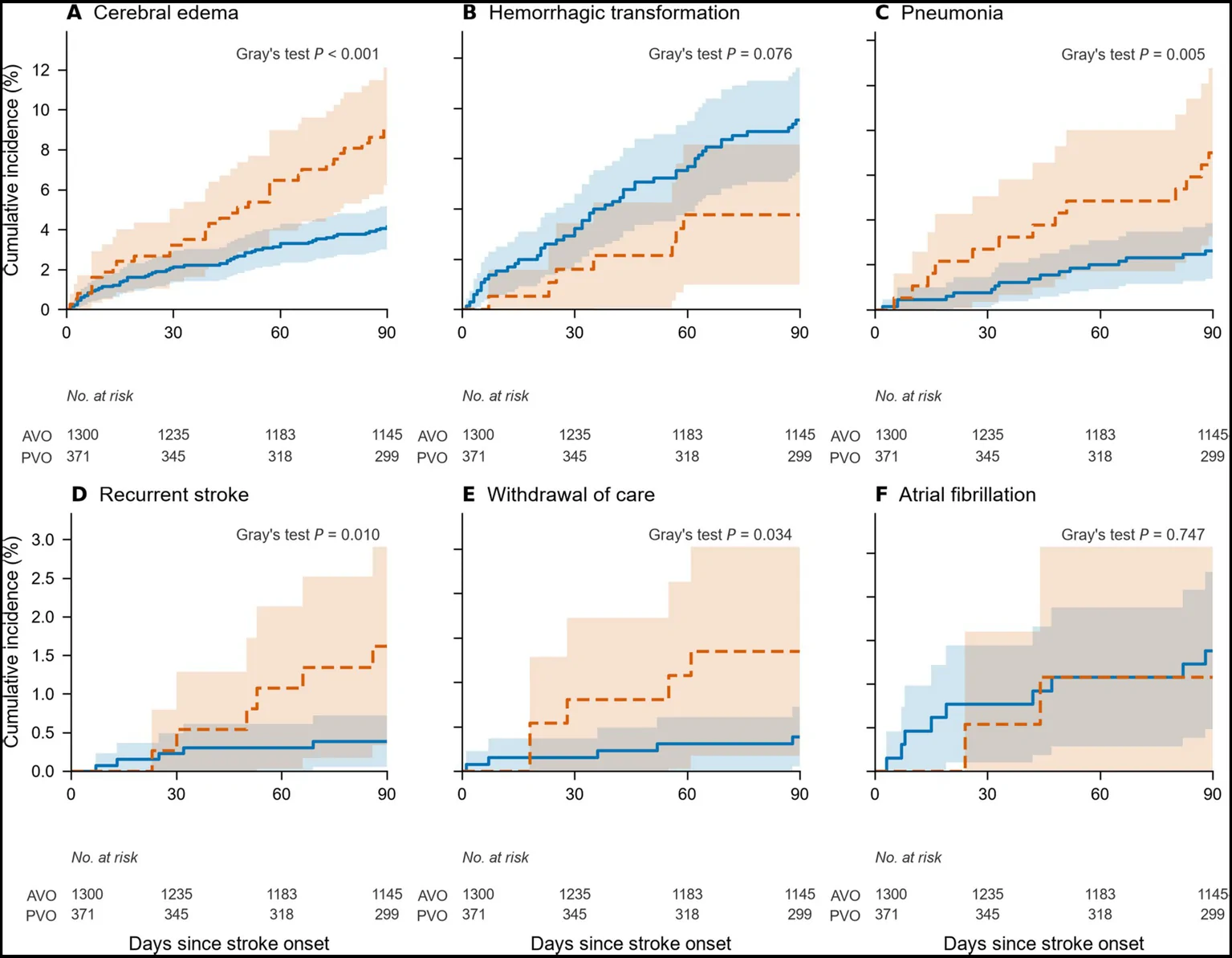
Cumulative incidence functions for 90-day cause-specific mortality, estimated by the Aalen-Johansen method in the complete cohort of 1,671 patients, with deaths from other causes treated as competing events. Panels show **(A)** cerebral edema, **(B)** hemorrhagic transformation, **(C)** pneumonia, **(D)** recurrent stroke, **(E)** withdrawal of care due to poor neurological prognosis, and **(F)** atrial fibrillation-related death. Solid blue lines denote anterior circulation occlusion (AVO); dashed orange lines denote posterior circulation occlusion (PVO); shaded bands represent 95% confidence intervals. AVO, anterior circulation large-vessel occlusion; PVO, posterior circulation large-vessel occlusion.

#### Cerebral edema

Cerebral edema-related death was significantly more frequent in PVO than in AVO. The 90-day cumulative incidence was 9.16% (95% CI 6.22–12.11) in PVO versus 4.15% (95% CI 3.07–5.24) in AVO (Gray’s test *p* < 0.001). In the Fine-Gray model, AVO was independently associated with a lower risk of cerebral edema-related death (adjusted sHR 0.47, 95% CI 0.30–0.73, *p* = 0.001; Benjamini-Hochberg adjusted *p* = 0.004). The finding was robust across all four sensitivity analyses (Model 1, sHR 0.42, 95% CI 0.27–0.65; Model 2, sHR 0.38, 95% CI 0.23–0.64; Model 3, sHR 0.48, 95% CI 0.30–0.78; Model 4 excluding withdrawal-of-care deaths, sHR 0.46, 95% CI 0.29–0.72) (Supplementary Table 2).

#### Hemorrhagic transformation

The 90-day cumulative incidence of fatal hemorrhagic transformation was 3.77% (95% CI 2.73–4.81) in AVO versus 1.89% (95% CI 0.50–3.27) in PVO (Gray’s test *p* = 0.076). In the Fine-Gray model, the point estimate indicated a higher risk in AVO, but the difference did not reach statistical significance (adjusted sHR 1.76, 95% CI 0.78–3.98, *p* = 0.17; Benjamini-Hochberg adjusted *p* = 0.267). This result was consistent across all sensitivity analyses (Model 1, sHR 1.81, 95% CI 0.81–4.01; Model 2, sHR 1.54, 95% CI 0.60–3.92; Model 3, sHR 1.42, 95% CI 0.63–3.20; Model 4, sHR 1.74, 95% CI 0.77–3.93) and in the extended model adjusted for TOAST classification, baseline NIHSS, and treatment times (sHR 2.75, 95% CI 0.89–8.47, *p* = 0.077) (Supplementary Table 3).

#### Other causes

Pneumonia-related death was more frequent in PVO (1.31% vs. 3.50%, adjusted sHR 0.34, 95% CI 0.17–0.68, *p* = 0.003), as were deaths from recurrent stroke (0.38% vs. 1.62%, adjusted sHR 0.29, 95% CI 0.10–0.86, *p* = 0.026) and from withdrawal of care due to poor neurological prognosis (0.38% vs. 1.35%, adjusted sHR 0.20, 95% CI 0.06–0.71, *p* = 0.013). Deaths attributed to atrial fibrillation (adjusted sHR 1.17, 95% CI 0.28–4.99, *p* = 0.83) and the composite cardiogenic category (adjusted sHR 0.78, 95% CI 0.31–1.93, *p* = 0.59) did not differ significantly between groups.

#### Composite endpoints

The neurogenic composite occurred in 8.69% of AVO patients and 14.02% of PVO patients (adjusted sHR 0.59, 95% CI 0.42–0.82, *p* = 0.002). The other-causes composite occurred in 1.54% of AVO and 3.77% of PVO patients (adjusted sHR 0.34, 95% CI 0.17–0.67, *p* = 0.002).

#### All-cause mortality

At 90 days, 228 patients (13.6%) had died: 156 (12.0%) in the AVO group and 72 (19.4%) in the PVO group (Table 3). After adjustment for the prespecified covariates, PVO remained independently associated with higher all-cause mortality (hazard ratio [HR] for AVO vs. PVO 0.56, 95% CI 0.42–0.74, *p* < 0.001). With additional adjustment for baseline NIHSS score, TOAST classification, and treatment times in the extended model, this association was attenuated and no longer statistically significant (HR 0.77, 95% CI 0.54–1.09, *p* = 0.14), indicating that much of the excess mortality in PVO is explained by its greater baseline stroke severity.

**Table 3:** All-cause mortality at 90 days.

Group	Deaths within 90 days, *n* (%)	Crude HR (95% CI)	*p*	Adjusted HR (95% CI)^1^	*p*	Fully adjusted HR (95% CI)^2^	*p*
AVO (*n* = 1,300)	156 (12.0)	0.60 (0.45–0.79)	<0.001	0.56 (0.42–0.74)	<0.001	0.77 (0.54–1.09)	0.14
PVO (*n* = 371)	72 (19.4)	Reference	NA	Reference	NA	Reference	NA

AVO, anterior circulation large-vessel occlusion; PVO, posterior circulation large-vessel occlusion; HR, hazard ratio; CI, confidence interval; NA, not applicable (reference category).

1 Cox proportional hazards model adjusted for age, sex, systolic blood pressure, type of anesthesia, history of atrial fibrillation, successful recanalization, and smoking status.

2 Cox proportional hazards model additionally adjusted for TOAST classification, baseline NIHSS score, onset-to-admission time, door-to-puncture time, puncture-to-recanalization time, and pretreatment intravenous thrombolysis (*n* = 1,499, complete cases; 172 patients excluded for missing covariate data, predominantly door-to-puncture time).

In a sensitivity analysis that added back the 45 deaths of unconfirmed cause, all-cause mortality was 14.4% in AVO and 21.1% in PVO (273 of 1716 patients, 15.9%), closely paralleling the estimates in the analytic cohort.

### Sensitivity analyses

Results were consistent across all four prespecified sensitivity analyses (Supplementary Table 2). The association between occlusion territory and cerebral edema-related death remained statistically significant in every model, and the association with hemorrhagic transformation remained non-significant in every model. In the extended model adjusted for TOAST classification, baseline NIHSS score, and treatment times, the edema association was attenuated to non-significance (adjusted sHR 0.85, 95% CI 0.48–1.52, *p* = 0.58), consistent with baseline stroke severity lying on the causal pathway between occlusion territory and fatal cerebral edema.

Adding stroke etiology (TOAST classification) to the model did not materially change any estimate (Supplementary Table 3): the hemorrhagic transformation estimate moved from 1.76 to 1.70, and the cerebral edema estimate from 0.47 to 0.45. Cardioembolic etiology was not associated with fatal hemorrhagic transformation in this cohort (sHR 1.09, 95% CI 0.41–2.93, *p* = 0.86, versus large-artery atherosclerosis) (Supplementary Table 4). The attenuation in the fully extended model was driven by baseline NIHSS score (sHR 1.07 per point, 95% CI 1.04–1.11, *p* < 0.0001 for cerebral edema) rather than by stroke etiology.

## Discussion

In this multicenter retrospective cohort of 1,671 Chinese patients with AIS-LVO who underwent EVT, we compared 90-day cause-specific mortality between AVO and PVO using competing-risk methods applied to the complete treated cohort. Three principal findings emerged. First, 90-day all-cause mortality was higher in PVO than in AVO (19.4% vs. 12.0%), although this difference was largely explained by greater baseline stroke severity. Second, cerebral edema-related death was significantly more frequent in PVO, a finding that was consistent across the four prespecified sensitivity analyses. Third, deaths from pneumonia and recurrent stroke were also more frequent in PVO, whereas fatal hemorrhagic transformation, although over-represented among anterior circulation deaths, did not show a statistically significant increase in risk in the complete cohort.

The predominance of cerebral edema as the leading cause of death in the PVO group reflects the anatomical and functional characteristics of the posterior intracranial circulation. The brainstem houses cardiorespiratory regulatory centers and the ascending reticular activating system, where even a small volume of vasogenic or cytotoxic edema can precipitate catastrophic and irreversible neurological deterioration (20). The significantly higher baseline NIHSS scores in our PVO cohort indicate more severe initial ischemic injury, which may trigger a maladaptive cascade of cytotoxic and vasogenic edema formation. The posterior fossa also imposes distinctive anatomical constraints: even moderate cerebellar or brainstem edema can cause fatal brainstem compression and obstructive hydrocephalus, which is the primary driver of early mortality in posterior circulation stroke (21).

The higher proportion of pneumonia-related deaths in the PVO group warrants consideration. Posterior circulation strokes frequently involve the lower cranial nerve nuclei, resulting in impaired bulbar function, dysphagia, and a markedly elevated risk of aspiration (22). Brainstem ischemia may also compromise the efferent cough reflex and central respiratory drive, further increasing susceptibility to severe aspiration pneumonia. The higher prevalence of current smoking in the PVO cohort may also increase the risk of adverse pulmonary complications in this population. These observations argue for routine dysphagia screening, early enteral feeding, and pulmonary hygiene in patients with PVO treated with EVT (23).

Fatal hemorrhagic transformation was over-represented among anterior circulation deaths. Although this pattern is numerically consistent with the established association between anterior circulation infarction and hemorrhagic complications after reperfusion, it did not translate into a statistically significant difference in risk at the population level. Hemorrhagic transformation accounted for 31.4% of anterior circulation deaths, but only 3.77% of anterior circulation patients died of it, compared with 1.89% of posterior circulation patients. The corresponding adjusted subdistribution hazard ratio was 1.76, with a 95% confidence interval that included unity, and the result was stable across all sensitivity analyses. Atrial fibrillation was markedly more prevalent in our AVO cohort (35.2% vs. 14.3%, *p* < 0.001). This difference has been proposed to increase hemorrhagic risk, because cardioembolic strokes show more severe blood–brain barrier disruption and a greater propensity for hemorrhagic transformation (24). Multiple clinical and imaging factors beyond occlusion territory have been associated with hemorrhagic transformation after reperfusion (25). The pooled evidence, however, does not support an excess of hemorrhage in patients with atrial fibrillation: in a meta-analysis of 18 observational studies comprising 16,096 patients, atrial fibrillation was not associated with symptomatic intracranial hemorrhage (OR 0.97, 95% CI 0.71–1.32), any intracranial hemorrhage (OR 1.08, 95% CI 0.91–1.28), or mortality (OR 0.92, 95% CI 0.79–1.08) after endovascular thrombectomy (26). Our finding of no statistically significant excess of fatal hemorrhagic transformation in AVO is consistent with that picture and suggests that the magnitude of any such effect is smaller than the crude distribution of causes among deaths would imply.

The two groups also differed in stroke etiology: cardioembolism was more frequent in AVO (37.3% vs. 18.3%), whereas large artery atherosclerosis was more frequent in PVO (65.5% vs. 47.2%). These differences are relevant to secondary prevention. Patients with atherosclerotic PVO may benefit from high-intensity statin therapy, strict blood pressure control, and antiplatelet regimens tailored to the underlying atherosclerotic disease (27). In contrast, the high burden of cardioembolic disease in deceased AVO patients argues for timely initiation of therapeutic anticoagulation and for management of concomitant heart failure in appropriately selected candidates (28). The higher rate of recurrent stroke-related mortality in the PVO group may reflect the diffuse, progressive nature of intracranial atherosclerosis in the vertebrobasilar circulation, which carries a substantial risk of early ischemic recurrence despite successful revascularization (29).

Our findings have clinical implications for the management of patients with AIS-LVO treated with EVT. First, risk stratification of patients being evaluated for EVT should incorporate circulation-specific risks of fatal post-procedural complications, alongside established determinants such as baseline blood pressure, which shows a J-shaped relationship with mortality after thrombectomy (30). For patients with PVO, management priorities should include earlier recognition of stroke to reduce onset-to-reperfusion times, proactive management of cerebral edema, and standardized pulmonary care to reduce the risk of fatal aspiration pneumonia. Second, the differences in underlying stroke etiology between AVO and PVO suggest that secondary prevention should be tailored to the individual patient’s stroke mechanism rather than applied uniformly to all patients with LVO.

This study has several limitations. First, its retrospective design and reliance on adjudicated causes of death without autopsy may have introduced misclassification. Although a prespecified adjudication hierarchy was applied to distinguish fatal hemorrhagic transformation from fatal cerebral edema, and cases in which the two mechanisms could not be separated were classified as indeterminate, residual misclassification remains possible. This applies particularly to hemorrhages of Heidelberg classes 1a–1c, which have limited mass effect and may coexist with extensive infarct-related edema. We could not re-adjudicate the original deaths retrospectively, because the source imaging was not available for central review; the hierarchy reduces this problem but does not eliminate it. Second, decisions regarding withdrawal of life-sustaining therapy varied across centers according to local practice, family preferences, and cultural factors, and may have masked the underlying neurological injury; excluding these deaths did not materially change the primary findings, but residual confounding by center remains possible. Third, 45 patients who died had no adjudicated cause of death and were excluded from the cause-specific analyses. Fourth, vessel-level occlusion site was unavailable in 39 patients (2.3%), and 122 patients were excluded from the analysis altogether, either because 90-day follow-up was missing (*n* = 77) or because the cause of death could not be confirmed (*n* = 45). Because neither group could contribute to the cause-specific analyses, the possibility that their exclusion influenced the estimates cannot be formally excluded. Fifth, although we adjusted for multiple prespecified confounders, unmeasured factors such as periprocedural hemodynamics and post-thrombectomy antithrombotic management could have influenced cause-specific mortality. Sixth, we deliberately present the primary model without adjustment for baseline NIHSS and TOAST classification, because both lie on the causal pathway between occlusion territory and fatal complications; the extended model that includes them is reported as a sensitivity analysis and attenuates the edema association. Seventh, the exclusively Chinese population with a high prevalence of intracranial atherosclerosis limits generalizability to other etiological profiles, and the small number of events for certain causes (for example, atrial fibrillation-related death) reduced statistical power. Finally, gastrointestinal hemorrhage could not be modeled separately because no events occurred in the posterior circulation group.

## Conclusion

Cause-specific mortality after EVT for AIS-LVO differs by vascular territory. Posterior circulation occlusion is independently associated with a higher risk of death from cerebral edema, pneumonia, and recurrent stroke, although the recurrent stroke association did not survive Bonferroni correction. Death from hemorrhagic transformation was over-represented among anterior circulation deaths, but this did not correspond to a statistically significant increase in risk when estimated in the complete treated cohort with competing risks accounted for. These findings support circulation-specific periprocedural strategies to improve survival after EVT and warrant prospective validation in future trials.

## Data Availability

The data analyzed in this study is subject to the following licenses/restrictions: data are not publicly available due to ethical restrictions imposed by the institutional review boards and the data-use agreement of the ANGEL-ACT registry, which protect patient privacy. Access requires formal approval from the ANGEL-ACT steering committee; interested researchers may contact the corresponding author to initiate the request process. Requests to access these datasets should be directed to dengyiming@bjtth.org.
