# Supplementary material for: Cause-specific mortality after endovascular treatment for anterior vs. posterior circulation large vessel occlusion: a multicenter cohort study
Source: Front Neurol. 2026 Oct 26;17:1948536. doi: 10.3389/fneur.2026.1948536 (PMC13745674; doi:10.3389/fneur.2026.1948536)
Supplement: Supplementary file 1 [file Table_1.docx]

**Supplementary Table 1. Baseline characteristics of the two groups of patients excluded from the analysis**

| **Characteristic** | **Lost to follow-up (n=77)** | **Unconfirmed cause of death (n=45)** | **P** |
| --- | --- | --- | --- |
| Age, years, median (IQR) | 68.0 (56.0–77.0) | 65.0 (55.0–73.0) | 0.143 |
| Male sex, % | 53.2 | 66.7 | 0.208 |
| Hypertension, % | 59.7 | 44.4 | 0.148 |
| Diabetes mellitus, % | 22.1 | 20.0 | 0.967 |
| Dyslipidemia, % | 6.5 | 4.4 | 0.947 |
| Atrial fibrillation, % | 41.6 | 42.2 | 1.000 |
| Current smoker, % | 15.6 | 20.0 | 0.708 |
| Systolic blood pressure, mmHg, median (IQR) | 145.0 (130.0–168.0) | 142.0 (130.0–160.0) | 0.252 |
| Baseline NIHSS, median (IQR) | 20.0 (16.0–28.0) | 20.0 (16.0–29.0) | 0.704 |
| ASPECTS, median (IQR)^a^ | 10.0 (8.0–10.0) | 9.0 (7.0–10.0) | 0.121 |
| Intravenous thrombolysis, % | 31.2 | 37.8 | 0.584 |
| Successful recanalization, % | 74.0 | 91.1 | 0.040 |
| General anesthesia, % | 48.1 | 42.2 | 0.663 |
| Onset-to-admission time, min, median (IQR)^b^ | 127.0 (54.2–201.8) | 169.0 (52.0–251.0) | 0.270 |
| Door-to-puncture time, min, median (IQR)^c^ | 127.0 (80.5–177.0) | 125.0 (80.0–169.2) | 0.934 |
| **Occlusion territory, n (%)** |  |  | 0.617 |
| Anterior circulation | 59 (76.6) | 37 (82.2) |  |
| Posterior circulation | 18 (23.4) | 8 (17.8) |  |
| **Vessel-level occlusion site, n (%)** |  |  | 0.886 |
| ICA | 24 (31.2) | 16 (35.6) |  |
| M1 | 31 (40.3) | 19 (42.2) |  |
| M2 | 4 (5.2) | 2 (4.4) |  |
| V-BA | 18 (23.4) | 8 (17.8) |  |

Categorical variables are compared with the chi-squared test. Missing data (n): ^a^ASPECTS, 4; ^b^onset-to-admission time, 1; ^c^door-to-puncture time, 12. All other variables were complete, and vessel-level occlusion site was recorded for all 122 patients. The two groups differed only in the rate of successful recanalization, which was lower among patients lost to follow-up.

Abbreviations: IQR, interquartile range; NIHSS, National Institutes of Health Stroke Scale; ASPECTS, Alberta Stroke Program Early CT Score; ICA, intracranial internal carotid artery; M1, first segment of the middle cerebral artery; M2, second segment of the middle cerebral artery; V-BA, vertebrobasilar.
