# Supplementary material for: Cause-specific mortality after endovascular treatment for anterior vs. posterior circulation large vessel occlusion: a multicenter cohort study
Source: Front Neurol. 2026 Oct 26;17:1948536. doi: 10.3389/fneur.2026.1948536 (PMC13745674; doi:10.3389/fneur.2026.1948536)
Supplement: Supplementary file 2 [file Table_2.docx]

**Supplementary Table 2. Sensitivity analyses of cause-specific mortality**

| **Cause of death** | **Model 1 sHR (95% CI)** | **P** | **Model 2 sHR (95% CI)** | **P** | **Model 3 sHR (95% CI)** | **P** | **Model 4 sHR (95% CI)** | **P** |
| --- | --- | --- | --- | --- | --- | --- | --- | --- |
| Cerebral edema | 0.42 (0.27–0.65) | <0.001 | 0.38 (0.23–0.64) | <0.001 | 0.48 (0.30–0.78) | 0.003 | 0.46 (0.29–0.72) | 0.001 |
| Hemorrhagic transformation | 1.81 (0.81–4.01) | 0.15 | 1.54 (0.60–3.92) | 0.37 | 1.42 (0.63–3.20) | 0.39 | 1.74 (0.77–3.93) | 0.18 |
| Pneumonia | 0.35 (0.18–0.71) | 0.004 | 0.23 (0.09–0.58) | 0.002 | 0.33 (0.15–0.70) | 0.004 | 0.33 (0.16–0.68) | 0.002 |
| Recurrent stroke | 0.24 (0.08–0.76) | 0.015 | 0.21 (0.05–0.93) | 0.04 | 0.31 (0.07–1.31) | 0.11 | 0.26 (0.09–0.80) | 0.019 |
| Atrial fibrillation | 1.26 (0.30–5.39) | 0.75 | 0.83 (0.16–4.40) | 0.82 | 0.96 (0.22–4.22) | 0.95 | 1.16 (0.27–4.92) | 0.84 |
| Withdrawal of care | 0.28 (0.08–0.98) | 0.046 | 0.09 (0.01–1.06) | 0.056 | 0.57 (0.14–2.36) | 0.43 | NE | NE |

Abbreviations: sHR, subdistribution hazard ratio; CI, confidence interval; NE, not estimable.
