# Supplementary material for: Cause-specific mortality after endovascular treatment for anterior vs. posterior circulation large vessel occlusion: a multicenter cohort study
Source: Front Neurol. 2026 Oct 26;17:1948536. doi: 10.3389/fneur.2026.1948536 (PMC13745674; doi:10.3389/fneur.2026.1948536)
Supplement: Supplementary file 3 [file Table_3.docx]

**Supplementary Table 3. Adjustment for stroke etiology and other baseline variables**

| **Cause of death** | **Base model sHR (95% CI)** | **P** | **Base + TOAST sHR (95% CI)** | **P** | **Fully extended model sHR (95% CI)** | **P** |
| --- | --- | --- | --- | --- | --- | --- |
| Cerebral edema | 0.47 (0.30–0.73) | 0.001 | 0.45 (0.29–0.71) | 0.001 | 0.85 (0.48–1.52) | 0.58 |
| Hemorrhagic transformation | 1.76 (0.78–3.98) | 0.17 | 1.70 (0.75–3.84) | 0.21 | 2.75 (0.89–8.47) | 0.077 |
| Pneumonia | 0.34 (0.16–0.68) | 0.003 | 0.32 (0.16–0.66) | 0.002 | 0.37 (0.17–0.82) | 0.014 |
| Recurrent stroke | 0.29 (0.10–0.86) | 0.026 | 0.28 (0.09–0.94) | 0.039 | 0.25 (0.08–0.78) | 0.017 |
| Atrial fibrillation | 1.17 (0.28–4.99) | 0.83 | 1.06 (0.24–4.66) | 0.94 | 1.45 (0.46–4.56) | 0.53 |
| Withdrawal of care | 0.20 (0.06–0.71) | 0.013 | 0.19 (0.05–0.68) | 0.011 | 0.19 (0.05–0.81) | 0.025 |

Base model: age, sex, systolic blood pressure, type of anesthesia, history of atrial fibrillation, successful recanalization, and smoking status. Base + TOAST: base model plus TOAST classification. Fully extended model: base model plus TOAST classification, baseline NIHSS score, onset-to-admission time, door-to-puncture time, puncture-to-recanalization time, and pretreatment intravenous thrombolysis. n = 1671 for the base and base + TOAST models; n = 1499 for the fully extended model (complete cases).

Abbreviations: CI, confidence interval; NIHSS, National Institutes of Health Stroke Scale; sHR, subdistribution hazard ratio; TOAST, Trial of Org 10172 in Acute Stroke Treatment.
