# Supplementary material for: Cause-specific mortality after endovascular treatment for anterior vs. posterior circulation large vessel occlusion: a multicenter cohort study
Source: Front Neurol. 2026 Oct 26;17:1948536. doi: 10.3389/fneur.2026.1948536 (PMC13745674; doi:10.3389/fneur.2026.1948536)
Supplement: Supplementary file 4 [file Table_4.docx]

**Supplementary Table 4. Etiology and severity terms in the fully extended model**

| **Covariate** | **Cerebral edema sHR (95% CI)** | **P** | **Hemorrhagic transformation sHR (95% CI)** | **P** |
| --- | --- | --- | --- | --- |
| TOAST: cardioembolic (vs large-artery atherosclerosis) | 0.96 (0.45–2.05) | 0.92 | 1.09 (0.41–2.93) | 0.86 |
| TOAST: other or undetermined etiology (vs large-artery atherosclerosis) | 2.80 (1.53–5.12) | 0.0009 | 3.55 (1.65–7.64) | 0.0012 |
| Baseline NIHSS, per point | 1.07 (1.04–1.11) | <0.0001 | 1.01 (0.97–1.05) | 0.58 |

Abbreviations: sHR, subdistribution hazard ratio; CI, confidence interval; NIHSS, National Institutes of Health Stroke Scale; TOAST, Trial of Org 10172 in Acute Stroke Treatment.
